# Plasma Level of Placenta-Derived Macrophage-Stimulating Protein -Chain in Preeclampsia before 20 Weeks of Pregnancy

**DOI:** 10.1371/journal.pone.0161626

**Published:** 2016-08-25

**Authors:** Yonggang Zhang, Hongling Yang, Yan Long, Qingling Ma, Ruihua Chen

**Affiliations:** 1 Department of Clinical Laboratory, Central Hospital of Longhua New District, Shenzhen, 518110, China; 2 Department of Clinical Laboratory, Guangzhou Women and Children’s Medical Center, Guangzhou Medical University, Guangzhou, 510623, China; 3 Department of Preventive Medicine, School of Public Health, Guangzhou Medical University, Guangzhou, 511436, China; University of Southampton, UNITED KINGDOM

## Abstract

**Object:**

This study aimed to investigate the diagnostic value of placenta-derived macrophage-stimulating protein α-chain (MSP-α) before the 20^th^ week of gestation for the early diagnosis of preeclampsia (PE).

**Methods and Materials:**

Two parts of this nested case-control study were simultaneously executed, and 1500 pregnant women were recruited. A total of 124 pregnant women were included in the plasma analysis part of this study. The MSP-α plasma level was measured before the 20^th^ week of gestation, and the participants were followed until delivery. A case group of 62 women with PE and a control group of 62 women matched by gestational age, maternal age, and pre-pregnancy BMI (with normotensive pregnancies) were evaluated. In the placenta analysis part of this nested case-control study, the placentas of 34 pregnant women were randomly obtained. The placental levels of MSP were measured in 17 individuals with PE (case group) and in 17 women with a normotensive pregnancy matched by gestational age and maternal age (control group).

**Results:**

The plasma level of MSP-α was higher in the PE group than in the control group before the 20^th^ week of gestation (p < 0.001). In addition, compared to the women with severe features in the PE group, those without severe features had a significantly higher plasma MSP-α level before the 20^th^ week of gestation (p < 0.001). The area under the receiver operating characteristic curve (AUC) of MSP-α before the 20^th^ week of gestation was 0.905 (95% CI, 0.811–0.962) for the women with early-onset PE without severe features. With regard to the placenta, the PE group (accumulated optical density, IOD [SUM] = 8862.37 ± 2064.42) exhibited increased MSP staining (more intense MSP staining or more extensive staining) compared with the control group (normal pregnancies (IOD [SUM] = 447.92 ± 114.72, P < 0.001). Furthermore, increased MSP staining was detected among the women without severe features compared with those with severe features in the PE group (IOD [SUM]: 12192.65 ± 5325.56 vs. 4104.83 ± 2383.06, P = 0.021).

**Conclusion:**

According to the findings of this study, the plasma level of MSP-α may be associated with PE, and MSP-α may be considered a candidate protein for further analysis in studies of PE. Multicenter studies with larger sample sizes must be performed in the future to obtain accurate results regarding the predictive value of MSP-α in combination with other protein factors for the early diagnosis of PE.

## Introduction

According to the ACOG criteria (2013), preeclampsia (PE) is defined by a systolic blood pressure ≥140 mmHg or a diastolic blood pressure ≥90 mmHg on two occasions measured at least 4 hours apart, with a proteinuria level ≥300 mg/d (or a minimum protein/creatinine ratio of 0.3 mg/dL or a proteinuria level ≥1+) or with severe features (≥1) in the absence of proteinuria after the 20^th^ week of gestation in women with previously normal blood pressure [1]. According to the ACOG (2013) criteria, PE is divided into PE without severe features and PE with severe features [1]. PE remains a major health issue affecting women and infants worldwide, and serious maternal-fetal morbidity and mortality still occur. The pathomechanism underlying the development of PE has not been clearly defined; nevertheless, a cascade of events, such as shallow placental implantation, poor placental perfusion, and adverse uterine spiral arterial remodeling [2], is known to play a pivotal role in the disrupting endothelial function in the maternal cardiovascular system. The placenta plays an essential role in the pathophysiology of PE, which begins to resolve upon delivery of the placenta [3]. It is necessary to identify early robust biomarkers to screen, diagnose, and monitor PE and to appropriately mitigate adverse outcomes, particularly in cases of severe PE [1]. Certain biochemical markers have been proposed for the early diagnosis of PE, including vascular endothelial growth factor (VEGF), soluble vascular endothelial growth factor receptor-1 (sFLT1), soluble endoglin (sENG) and placental growth factor (PIGF) [4]. To date, however, there is no single factor that can independently and effectively predict PE. Thus, it may be useful to identify additional plasma proteins that can be used as predictive biomarkers of PE in a future prediction model study. Another marker of interest is receptor tyrosine kinase Ron (RON), which regulates cell growth, differentiation, and morphology [5]. RON is also a potential trophic factor that contributes to the proliferation and migration of cells in various tissues [5]. Macrophage-stimulating protein (MSP), the ligand of RON, is also called hepatocyte growth factor receptor-like (HGFL) protein; it is composed of an alpha chain (60 kDa) and a beta chain (31 kDa) that are linked by disulfide bonds. Primary MSP may be released from the membranes of liver cells through proteolysis, giving rise to MSP-α, which binds to RON and activates it for regulation of its target. Recent studies have indicated that RON is involved in placental implantation and trophoblast function and viability [6] and that Ron -/- embryos are viable through the blastocyst stage of development; however, they fail to survive beyond the peri-implantation period [7]. RON is expressed in the invading ectoplacental cone and trophoblast giant cells. surrounding the implanting embryo during pregnancy, and it assumes a paracrine role in trophoblasts, which express its ligand, MSP [6]. It has been demonstrated that activation of RON by HGFL/MSP may assist in implantation, thereby improving trophoblast function and viability [6]. Our previous preliminary study has revealed that the plasma level of MSP is abnormal in pregnancy-induced hypertension. The presence of an abnormal plasma level of MSP-α has not been found to be useful for the early diagnosis of PE before clinical symptoms arise. PE is generally considered the most common disease and the cause of death among pregnant women and fetuses; however, no appropriate early biomarkers have been identified with sufficient predictive capacities. We investigated the diagnostic value of the placental-derived MSP-α level before the 20^th^ week of gestation for early detection of PE. Furthermore, we analyzed the diagnostic efficacy of this marker in PE with severe features, PE without severe features, early-onset PE and late-onset PE.

## Materials and Methods

**Fig 1:**
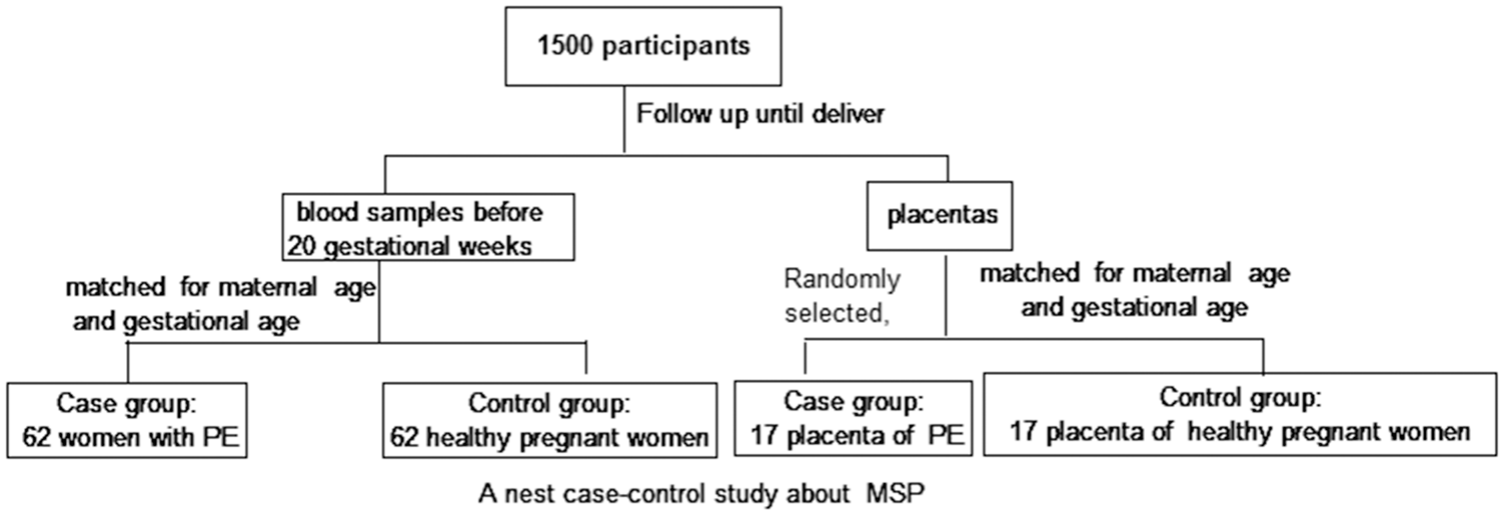
The study design.

A nested case-control study of plasma MSP-α in pregnant women with or without PE and a comparison of MSP protein expression in placenta specimens from pregnancies with or without PE were performed at the Guangzhou Women and Children’s Medical Center, China, between Feb 2014 and May 2015. In the nested case-control study, these pregnant women were followed until delivery. The women who subsequently developed PE and delivered at the hospital formed the case group and were then further classified as having early-onset (<34 weeks) or late-onset PE (≥34 weeks), as well as PE with or without severe features (Severe features of preeclampsia; S1 Text). The control group consisted of pregnant women without hypertension followed during the same study period, and these women were matched with the women in the case group according to the maternal age, gestational age, and pre-pregnant body mass index (BMI). Additional details of the study design are provided in Fig 1.

### Patients and sample collection

The Human Research Ethics Committee of Guangzhou Women and Children’s Medical Center of Guangzhou Medical University approved this study (approval number: 2013082619), and all participants provided written informed consent and completed a questionnaire. In total, 1500 pregnant women were recruited for this study. Women at 8–20 weeks of pregnancy were eligible for inclusion; these women were referred to the Guangzhou Women and Children’s Medical Center for collection of a 2 cc blood sample in an EDTA anticoagulation tube. The plasma was separated by centrifugation for 10 minutes at 3000 rpm and then stored at −76°C. The pregnant women were followed until they delivered (by Cesarean section or spontaneous delivery, as shown in S1 Table), and their placentas were obtained. The decidual tissue was not removed, and multiple sites in one placenta were randomly selected. Then, the placental tissues (including the decidual tissue) were formalin fixed, paraffin embedded and preserved in the placenta bank of a clinical biological resource bank. All PE and normal pregnancies were diagnosed by a senior obstetrics and gynecology specialist according to the 2013 ACOG criteria.

### Sample selection and ELISA array

**Fig 2:**
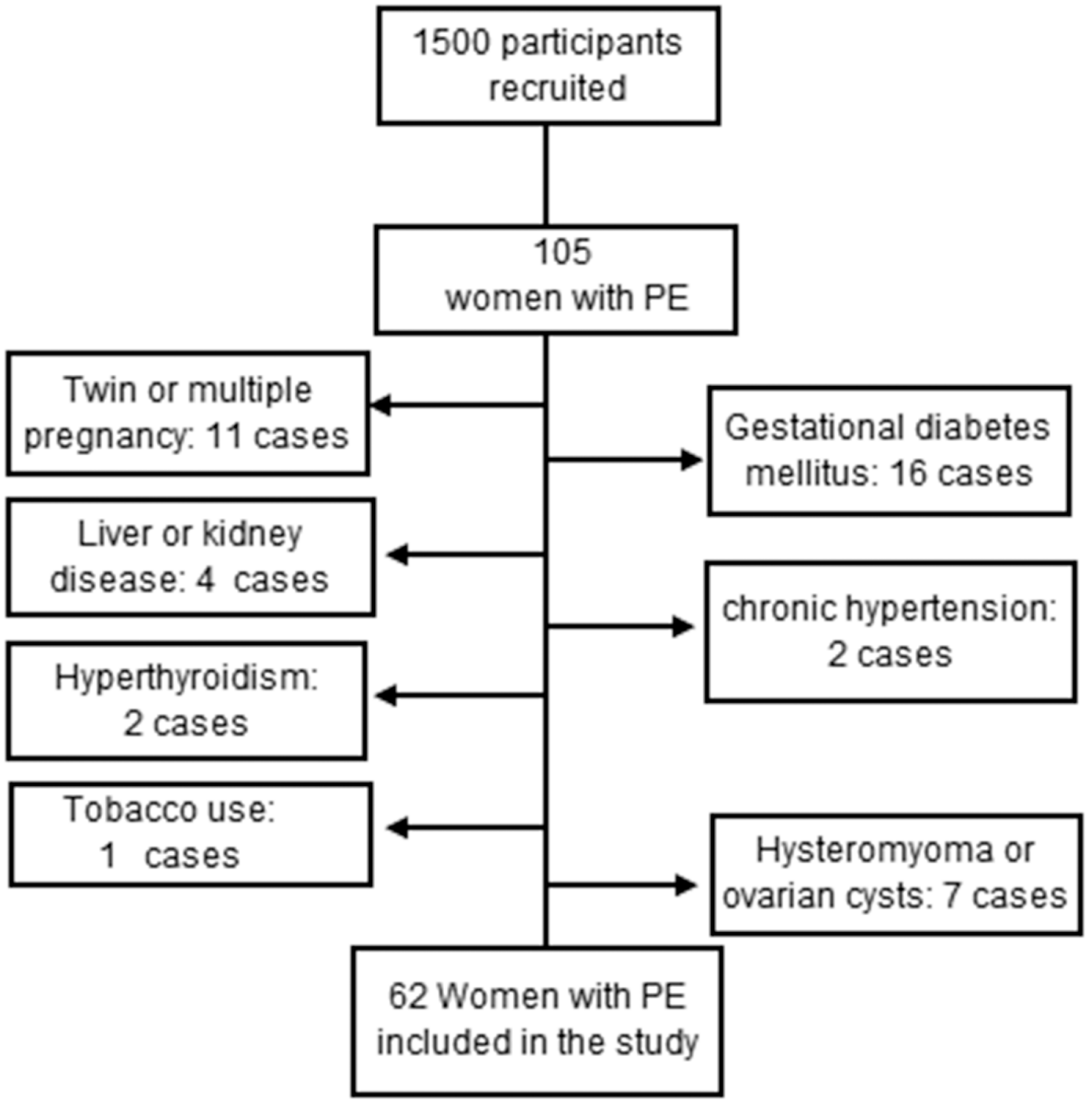
A flow diagram of the participants in the plasma MSP study.

The plasma samples consisted of 62 case and 62 control samples selected from among the samples provided by the 1500 pregnant women. The following exclusion criteria were applied (Fig 2): liver and renal disease, chronic hypertension, multiple pregnancy, gestational diabetes mellitus, autoimmune disease and tobacco use. The cases and controls were matched by maternal age, gestational age, and pre-pregnancy BMI. The clinical characteristics are shown in Table 1. The plasma levels of MSP-α in both groups were measured using the blinded method (by unknown technicians) with RAYBIO ELISA kits (RayBiotech, Inc., Norcross, GA, USA). Briefly, ELISA was performed in accordance with the manufacturer's instructions. A gradient solution was prepared to determine the possible concentration range of MSP-α, and two standard curves were generated. The absorbance values of all duplicate specimens were measured by ELISA and converted to the corresponding concentrations using the standard curves. The absorbance values were determined for each well using a Bio-Tek Elx800 microplate reader (BioTek, Inc. Winooski, VT, USA); standard curves were constructed and concentrations were determined using Sigma Plot software package (Systat Software, Inc. San Jose, CA, USA).

**Table 1:** Demographic and Clinical Characteristics of the PE and Control Groups.

Characteristic	Case (PE)	Control	*P-*Value
Gestational age at sample collection (week)	14.63±3.39	14.79 ±3.25	0.787
Maternal age (year)	29.92±4.38	29.00±3.80	0.383
Pre-pregnancy BMI (kg/m^2^)	21.47±2.81	20.04±2.29	0.085
MSP (ng/mL before the 20^th^ week of gestation	172.78±57.38	131.84±56.47	<0.001
Gestational age at delivery (weeks)	35.89±3.84	39.13±1.41	<0.001
Systolic (mmHg)	153.54±13.86	116.48±10.55	<0.001
Diastolic (mmHg)	101.37±9.12	75.03±7.90	<0.001
Proteinuria (g/24 h)	3.05±4.65	0	0
Thrombocytopenia (<1×10^5^/mL) (n, %)	2 (3.23%)	0	<0.05
Newborn weight (g, n)	2536.69±700.82(59)	3225.4±392.72(62)	<0.001
SGA newborn (n, %)	27 (43.55%)	3 (4.84%)	<0.05
FGR (n, %)	10 (16.13%)	0 (0)	<0.05
Cesarean section, forceps delivery or induced labor (n, %)	45 (72.6%)	7 (11.3%)	<0.05
Stillbirth (n)	3 (4.84%)	0 (0)	<0.05
MSP (ng/ml) at (8–12) weeks	152.95±53.44	120.09±54.56	0.024
MSP (ng/ml) at (13–16) weeks	180.74±62.45	141.18±60.45	0.03
MSP (ng/ml) at (17–20) weeks	176.29±55.84	132.26±54.26	0.024

BMI: Body mass index, SGA: Small for gestational age (weight of below the 10^th^ percentile for gestational age), FGR: Fetal growth restriction

### Immunohistochemistry

Seventeen paired placentas (i.e., 17 PEs and 17 controls) were randomly selected from among the nested cohort specimens and screened for the aforementioned exclusion criteria. The case and control groups were paired at a 1:1 ratio by maternal and gestational age. The placental tissues were randomly selected and then were longitudinally sectioned. The placental sections included all components of the placenta. Finally, the sections were selected for preparation using the blind method.

MSP and RON were detected using a rabbit anti-human MSP or RON antibody (catalog no. [EPR6207] ab124787; catalog no. [EP1132Y] ab52927; Abcam, Cambridge County, UK) and an anti-rabbit cell and tissue staining kit, REAL^™^ EnVision+/HRP RABBIT (catalog no. K5007; Dako Denmark A/S, Denmark), as previously described with modifications [8]. Antigen retrieval was performed using EDTA buffer and microwave heating. Staining for MSP was based on formation of a horseradish peroxidase (HRP) and anti-rabbit antibody (2^nd^ antibody) complex, bound to a rabbit anti-human antibody (1^st^ antibody) targeting MSP or RON. Visualization was based on enzymatic conversion of a chromogenic substrate, 3,3’-diaminobenzidine (DAB), into a brown precipitate by horseradish peroxidase at sites of MSP or RON antigen localization. A separate antibody preparation with an MSP or RON rabbit anti-human antibody (1:50 dilution) was made for each sample. These preparations were generated with blinding (no knowledge of the identities of the samples). Digital photomicrographs were obtained for use in grading of the identified normal and PE sections. The stained sections were evaluated for MSP and RON expression by 2 investigators who were blinded to the histologic diagnoses using Image Pro Plus 6.0 software (Media Cybernetics, Inc. Rockville, MD, USA). The accumulated optical density and the corresponding brown-yellow positive area were provided with each photomicrograph. Finally, each group was represented by the accumulated optical density (IOD [SUM]).

### Statistical analysis

In plasma analysis, SPSS Statistics software, version 19.0 (IBM, Chicago, IL), was used to assess the clinical characteristics, continuous variables were summarized using the mean and standard deviation, and categorical variables were summarized using numbers and percentages. Study subgroups were compared using the independent sample T-test or Mann-Whitney U-test for continuous data (to determine normality) and Fisher’s exact test for categorical data. The reported P values are 2-sided, and a P < 0.05 was considered statistically significant. Medcalc v9.2.0.1 (MedCalc Software bvba, Ostend, Belgium) was used to generate receiver operating characteristic (ROC) curves, and the sensitivity, specificity, positive predictive value (PPV), negative predictive value (NPV), likelihood ratio, relative risk and area under the ROC curve (AUC) were determined. The data are presented as the mean and 95% confidence interval.

In immunohistochemical analysis, all slices in each group were magnified 200 times using a microscope (Leica4000B, Leica Instrument Co., Ltd., Hong Kong), and 3 microscopic fields were randomly selected. Fields were selected that contained the maximum amount of placental tissues, and the background light in each photo was kept consistent. Image Pro-Plus 6.0 software was used to determine the integrated optical densities (IODs) of the MSP-positive reactions, i.e., the absorbance values of yellow or brownish-yellow stain, which were significantly higher than the background values. SPSS software (version 19.0) was used for analysis of significant differences. The data are presented as the mean ± standard error.

## Results

### Plasma MSP

The results indicated that women who developed PE later had significantly higher plasma MSP-α levels before the 20^th^ week of gestation compared to those in the control group (p<0.001). The demographics of the patients in the case and control groups are provided in Tables 1 and 2. The delivery methods also significantly differed between the two groups. In the case group, 45 patients (72.6%) required C-section, forceps delivery or induced labor, whereas only 7 patients (11.3%) in the control group required C-sections.

**Table 2:** Demographic and Clinical Characteristics of PE who are grouped in early onset PE, late onset PE, “without severe features” and “with severe features”.

Characteristic	Later onset (n = 43)	Early onset(n = 19)	With severe features(n = 27)	Without severe features(n = 35)
Maternal Age(year)	29.37±4.07	31.16±4.90	29.44±4.28	30.29±4.48
Pre-pregnancy BMI (kg/m2)	21.74±2.75	20.88±2.93	21.29±2.88	21.62±2.80
MSP (ng /ml) before 20 weeks of gestation	168.42±57.87	197.43±61.49	141.61± 51.01	204.86 ± 51.79
Gestational age at delivery(week)	37.88±1.64	31.38±3.54	33.61±4.33	37.65±2.17
Systolic(mmHg)	150.91±13.81	158.95±14.71	165.67±8.95	143.89±10.08
Diastolic(mmHg)	100.30±9.92	102.95±8.51	108.48±7.62	95.43±6.47
Proteinuria(g/24h)	2.24±2.20	4.90±7.53	5.06±6.42	1.50±1.29
newborn-weight(g, n)	2833.95±492.16	1545.53±669.62	1949.07±816.95	2817.14±576.03
Cesarean or section Forceps delivery or induced labor (n, %)	26(60.5%)	19(100%)	27(100%)	18(51.4%)
Stillbirth (n)	0 (0%)	3 (%)	3 (%)	0 (0%)

There are total 19 early onset PE, of which 11 (58%)had severe features, 8 (42%)is “without severe features”; and there are total 43 late onset PE, of which 16 (37%)had severe features, 27 (63%) is “without severe features”.

**Fig 3:**
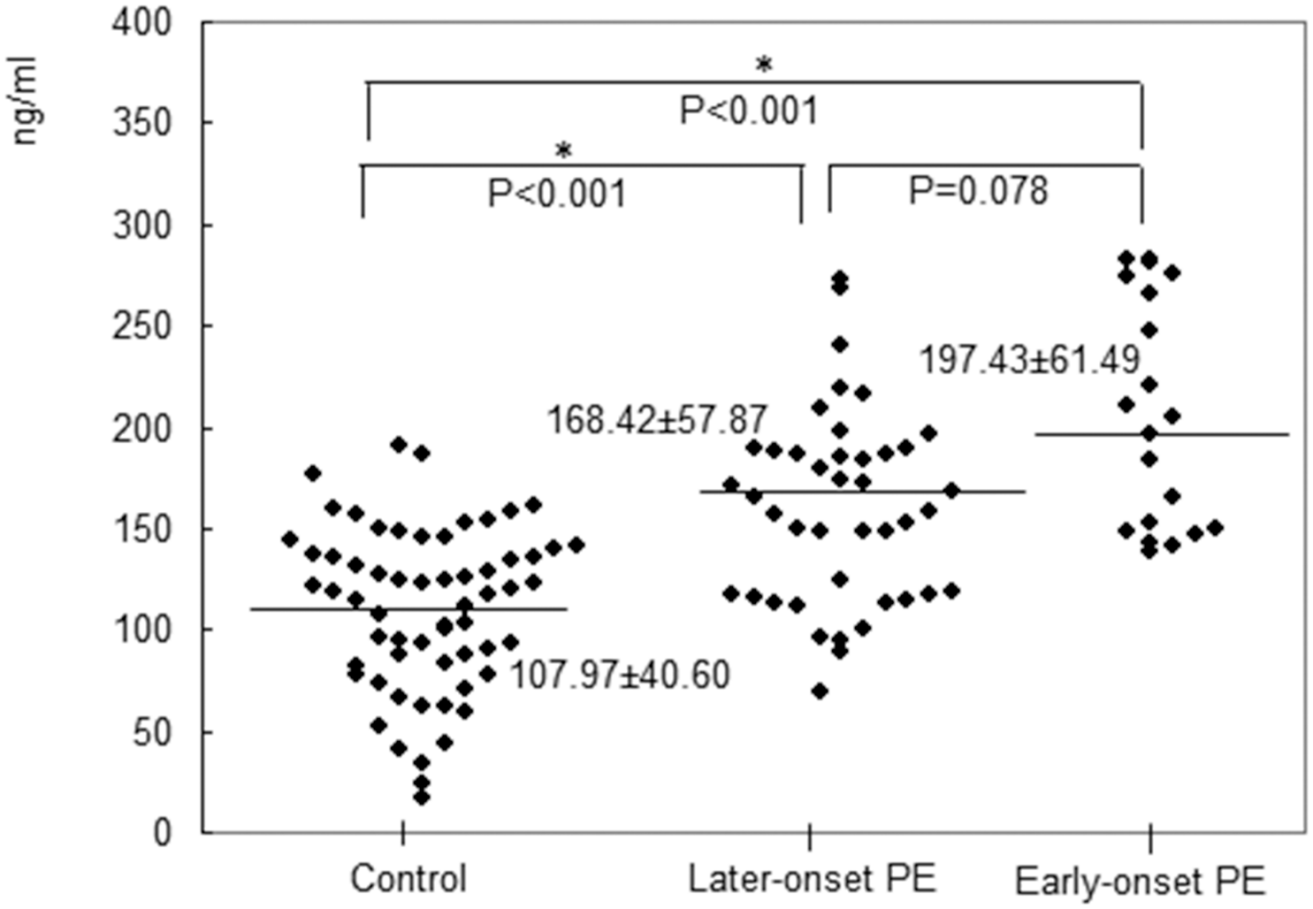
The plasma level of MSP-α was significantly higher in the patients with early-onset preeclampsia (197.43± 61.49 ng/mL) compared to the controls (107.97± 40.60 ng/mL) before the 20^th^ week of gestation (p<0.001). Before 20 weeks of pregnancy, significant differences in the plasma levels of MSP-α between the patients with late-onset preeclampsia and the controls were observed (168.42± 57.87 ng/mL vs. 107.97± 40.60 ng/mL, p < 0.001).

**Fig 4:**
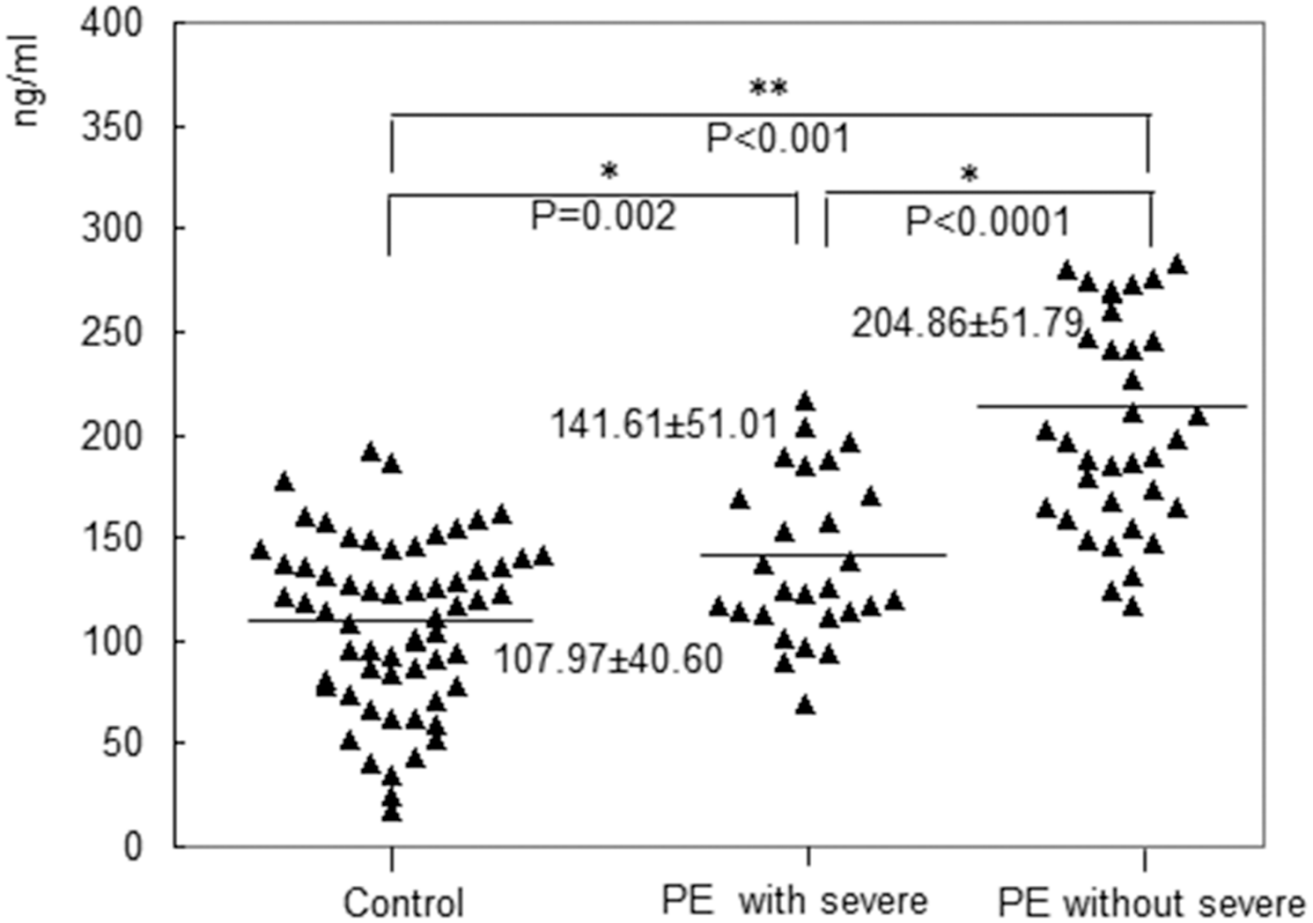
The plasma level of MSP-α in the patients with severe features was significantly lower than that in the patients without severe features before the 20^th^ week of gestation (141.61 ± 51.01 ng/mL vs. 204.86 ± 51.79 ng/mL, p < 0.001). The levels in the case groups were significantly higher compared with that in the control group (204.86 ± 51.79 ng/mL vs. 141.61 ± 51.01 ng/mL vs. 107.97± 40.60 ng/mL, p < 0.001).

The case group consisted of 62 PE cases, including 19 (30.6%) early-onset (<34 weeks) cases and 43 (69.4%) late-onset (≥34 weeks) cases. A total of 11 of the early-onset cases and 16 of the late-onset cases had severe features. There was no significant difference in the MSP-α levels between the early- and late-onset cases (p = 0.078). However, before the 20^th^ week of gestation, the plasma level of MSP-α was significantly higher in the patients with early-onset PE (197.43 ± 61.49 ng/mL) compared to the controls (107.97 ± 40.60 ng/mL, p < 0.001). Furthermore, before the 20^th^ week of gestation, significant differences in the plasma levels of MSP-α were observed between the late-onset PE group and control group (168.42 ± 57.87 ng/mL vs. 107.97 ± 40.60 ng/mL, p < 0.001). These findings are presented in Fig 3. In the case group, which consisted of 62 PE patients, 35 patients (56.5%) had PE without severe features, and 27 (43.5%) had PE with severe features. Before the 20^th^ week of gestation, the plasma level of MSP-α was significantly lower in the patients with severe features compared to those without severe features (141.61 ± 51.01 ng/mL vs. 204.86 ± 51.79 ng/mL, p < 0.001). Furthermore, the levels of MSP-α in both case groups were significantly higher than that in the control group (204.86 ± 51.79 ng/mL vs. 141.61 ± 51.01 ng/mL vs. 107.97 ± 40.60 ng/mL, p < 0.001). These findings are presented in Fig 4.

Table 3 shows several AUCs, as well as the sensitivities, specificities, positive predictive values, negative predictive values, positive likelihood ratios, negative likelihood ratios and risk ratios of MSP-α before the 20^th^ week of gestation for early-onset PE without severe features and early-onset PE with severe features, in addition to late-onset PE with or without severe features. MSP measurement was more effective for predicting the early-onset PE with severe features (AUC = 0.708, 95% CI, 0.590–0.809) than the late-onset PE with severe features (AUC = 0.599, 95% CI, 0.482–0.708) before the 20^th^ week of gestation. In addition, the AUC for MSP was higher for early-onset PE without severe features than for late-onset PE without severe features before the 20^th^ week of gestation (0.905, 95% CI, 0.811–0.962 vs. 0.825, 95% CI, 0.730–0.897). Further, MSP is a stronger diagnostic indicator of PE without severe features than PE with severe features accompanying early-onset or late-onset disease.

**Table 3:** The cut-off values and diagnostic power of plasma MSP-α before the 20^th^ gestational week for typing preeclampsia.

PE	GW-c	Cutoff point	Sensitivity (%)	Specificity (%)	PPV (%)	NPV (%)	RR	PLR	NLR	AUC	P -Value
			95% CI	95% CI	95% CI	95% CI		95% CI	95% CI	95% CI	
Late onset with severe features	8–20 weeks	< = 119.4082	81.25	62.9	36.1	92.9	5.1	2.19	0.3	0.599	0.1995
			54.4–96.0	49.7–74.8	20.8–53.8	80.5–98.5		1.6–3.0	0.1–0.9	0.482–0.708	
Early onset with severe features	8–20 weeks	>139.6048	81.82	61.29	27.3	95	5.5	2.11	0.3	0.708	0.0078
			48.2–97.7	48.1–73.4	13.3–45.5	83.1–99.4		1.5–3.0	0.08–1.1	0.590–0.809	
Late onset without severe features	8–20 weeks	>146.2706	92.59	67.74	55.6	95.5	12.2	2.87	0.11	0.825	0.0001
			75.7–99.1	54.7–79.1	40.0–70.4	84.3–99.5		2.3–3.5	0.03–0.4	0.730–0.897	
Early onset without severe features	8–20 weeks	>161.8021	87.5	79.03	35	98	17.5	4.17	0.16	0.905	0.0001
			47.3–99.7	66.8–88.3	15.4–59.2	89.4–99.9		3.1–5.6	0.02–1.1	0.811–0.962	

GW-c: gestational weeks at samples collection; AUC: area under the receiver operating characteristic curve; PPV: positive predictive value; NPV: negative predictive value; PLR: positive likelihood ratio; NLR: negative likelihood ratio; RR: relative risk or risk ratio; early onset: less than 34 weeks of gestation; late onset: 34 weeks of gestation or greater

The highest AUC (0.905, 95% CI, 0.811–0.962) and Youden Index (sensitivity [87.50%] + specificity [79.03%]—1) were associated with a threshold of 161.80 ng/mL before the 20^th^ week of gestation for early-onset PE without severe features. The second highest AUC (0.825, 95% CI, 0.730–0.897) and Youden Index (sensitivity [92.59%] + specificity [67.74%] -1) were associated with a threshold of 146.27 ng/mL before the 20^th^ week of gestation for late-onset PE without severe features.

**Fig 5:**
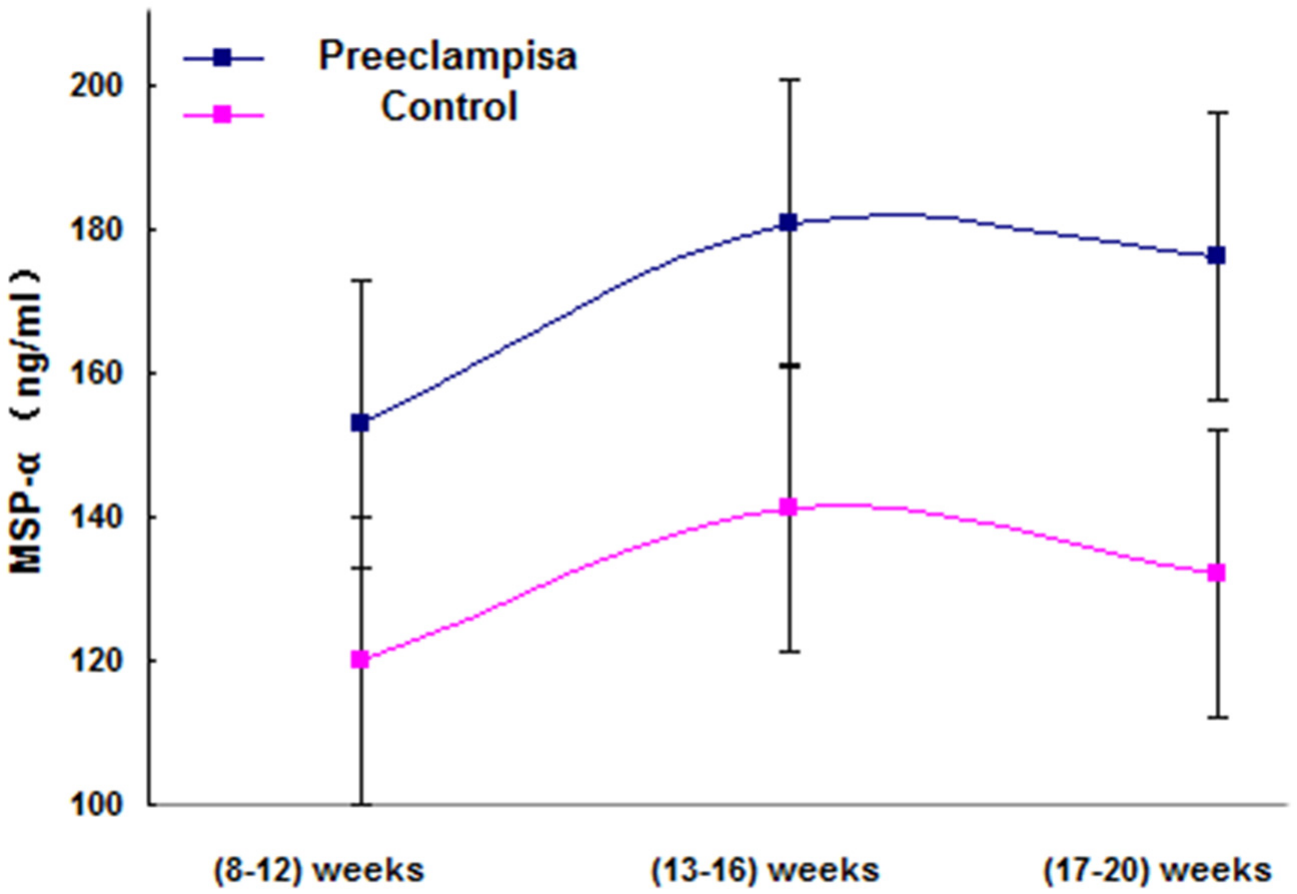
In women with preeclampsia and in those with normal pregnancy, before the 20^th^ week of gestation, fluctuations in the MSP-α level were examined at three time points (8–12, 13–16, and 17–20 gestational weeks), revealing that the mean (±SE) levels of MSP-α (in the weeks prior to the 20^th^ week of gestation) in each group did not significantly differ.

In the group of women with PE, the MSP-α levels fluctuated prior to the 20^th^ week of gestation at three time points (i.e., 8–12, 13–16, and 17–20 gestational weeks; three subgroups), with no significant differences in the mean levels among the subgroups (p > 0.05) (Fig 5). Similar findings were observed in the group of normal pregnant women (Fig 5).

### Placental MSP

**Fig 6:**
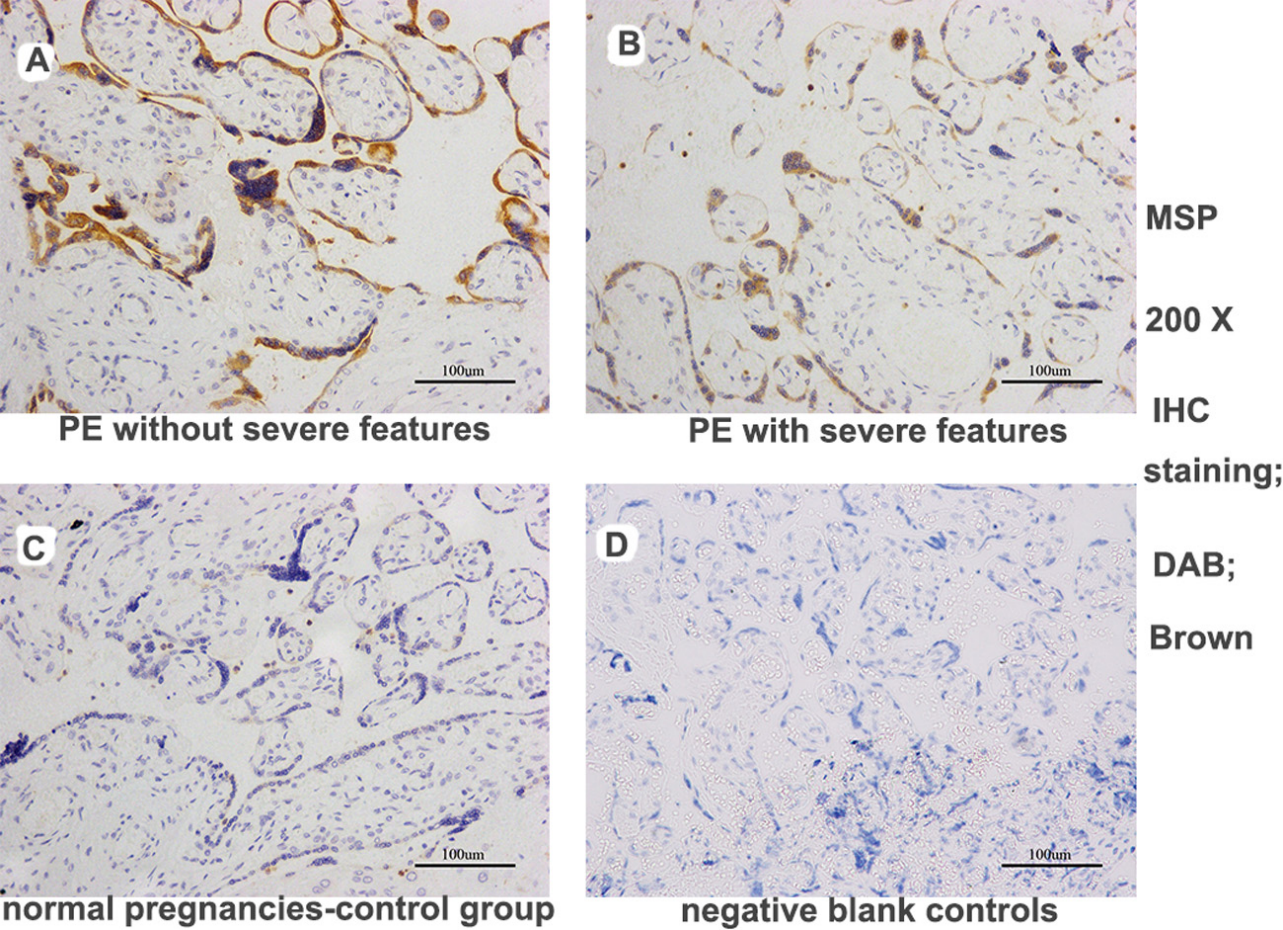
MSP expression in trophoblast cells in placental sections from pregnant women, as determined by immunohistochemical staining. The PE group exhibited strong MSP staining in the placenta compared with the control group. Furthermore, increased MSP staining was detected in the sections from the patients without severe features vs. those with severe features in the PE group (DAB, brown: images A-D, 200X magnification). (images A: PE without severe features; images B: PE with severe features; images C: normal pregnancies in the control group; images D: negative blank controls).

**Fig 7:**
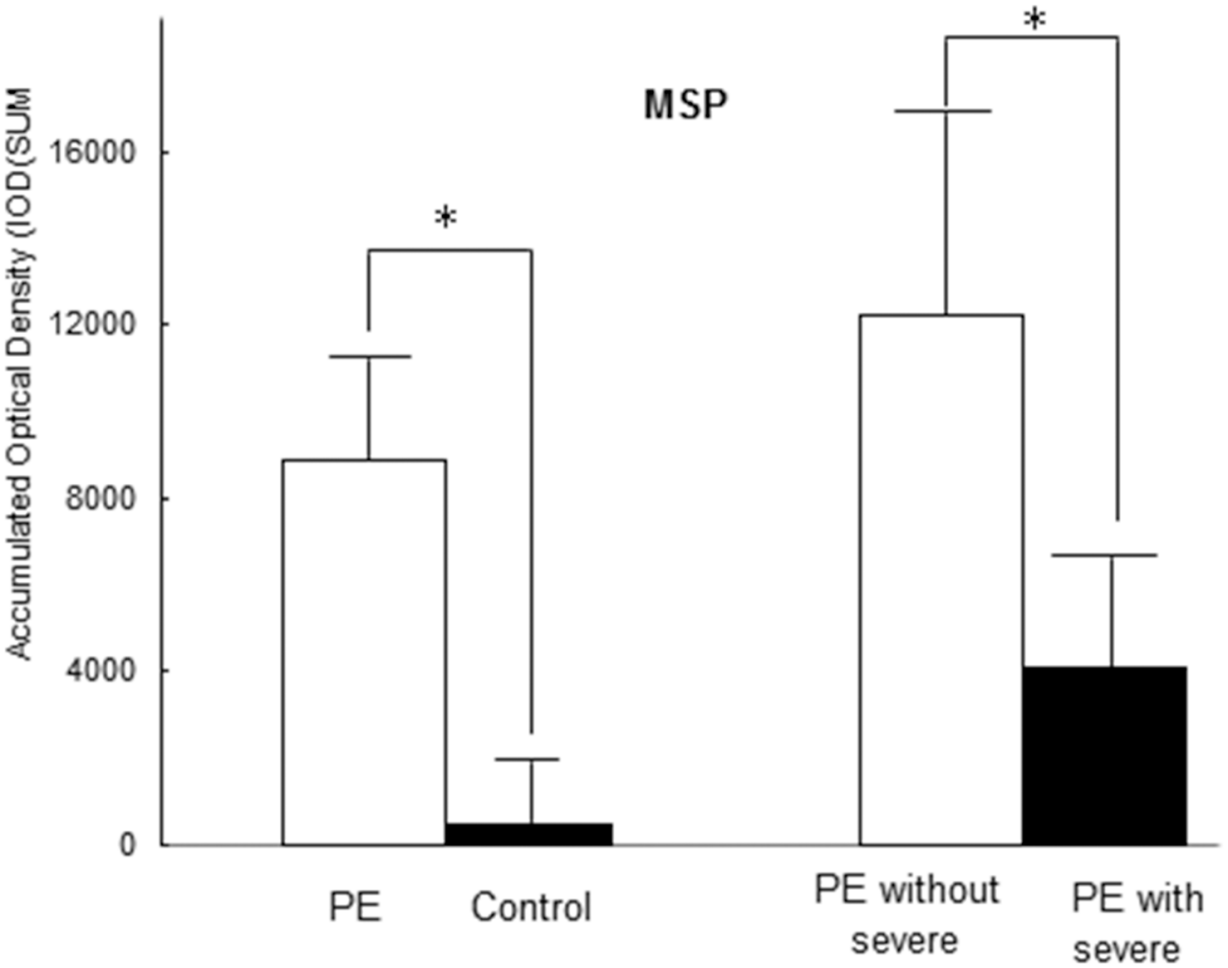
Immunohistochemical analysis of the placentas revealed that the patients with PE exhibited significantly increased plasma MSP staining (IOD [SUM] = 8862.37±2064.42) compared with the healthy pregnant controls (IOD [SUM] = 447.92±114.72, P <0.001). A significant difference in MSP staining was observed between the PE group without severe features (IOD [SUM]: 12192.65±5325.56) and the PE group with severe features (4104.83±2383.06; P = 0.021).

**Fig 8:**
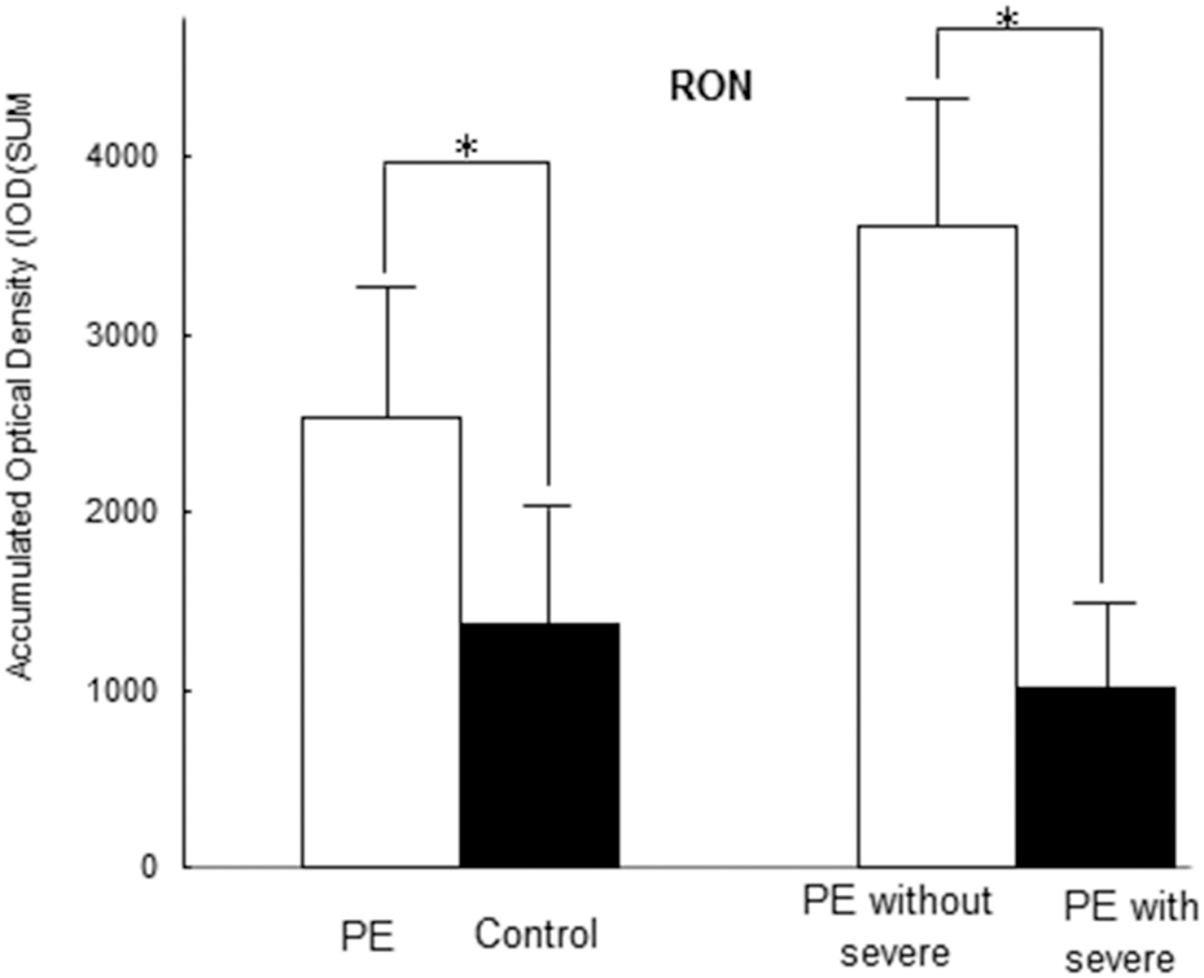
Immunohistochemical analysis of the placentas revealed that the patients with PE exhibited significantly increased plasma RON staining (IOD [SUM] = 2540.15±637.76) compared with the healthy pregnant controls (IOD [SUM] = 1375.87±365.03, P <0.001). A significant difference in RON staining was detected between the PE group without severe features (IOD (SUM):3611.78±1020.86) and the PE group with severe features (1009.25±158.36; P = 0.017).

Seventeen paired cases and controls were available for analysis. The PE placentas in the case group exhibited increased MSP staining (more intense or more extensive MSP staining) (IOD [SUM] = 8862.37 ± 2064.72) compared with the normal control placentas (IOD [SUM = 447.92 ± 114.72, P < 0.001). Furthermore, increased MSP staining was observed in the PE group without severe features compared with the PE group with severe features (IOD [SUM]: 12192.65 ± 5325.56 vs. 4104.83 ± 2383.06, P = 0.021), as shown in Fig 6 (images A-D) and Fig 7. The RON staining results were in agreement with the MSP results (S1 Fig, images E-G, and Fig 8); RON staining was increased in the PE group (IOD [SUM] = 2540.15 ± 637.76) compared with the healthy pregnancy control group (IOD [SUM] = 1375.87 ± 365.03, P <0.001). The RON staining pattern significantly differed between the PE group without severe features (IOD [SUM]:3611.78 ± 1020.86) and the PE group with severe features (1009.25 ± 158.36; P = 0.017) (S1 Fig, images E-G, and Fig 8).

## Discussion

Although the pathogenesis of PE is not completely understood, most scholars believe that a lack of placental perfusion is the main factor underlying its occurrence [9]. Further study of the pathophysiology of the PE placenta has revealed that the two main pathological characteristics in early pregnancy are disordered remodeling of the uterus spiral arteries and decreased invasion of extravillous trophoblast cells [10]. Specifically, because of maternal and fetal factors, the invasion of placental extravillous trophoblasts is deficient, vascular remodeling is poor, and the placenta is hypoperfused, hypoxic and ischemic, all of which lead to the increase of oxidative stress in the placenta and the secretion of vasoactive substances by trophoblast cells (e.g., VEGF; sFlt-1, endoglin, etc.) in the placenta. These substances enter into the maternal circulation, resulting in the endothelial cell injury and a series of clinical manifestations of PE [11].

Here, we confirmed that the plasma level of MSP-α was significantly higher in pregnant women with PE than in those with a normal pregnancy before the 20^th^ week gestation. These results suggest that MSP-α might be involved in the pathogenesis of PE. In addition, we found that early-onset PE without severe features was most strongly correlated with the MSP-α level, corresponding to the highest level, in direct contradiction to the findings for late-onset PE with severe features. These results might indicate that MSP is a favorable factor for development of PE. Considering that the MSP-α level was a positive factor for PE, its increase in the case group compared with the control group was an unexpected finding. The elevation of the MSP-α level may be a stress reaction to PE, and MSP-α may confer protection against PE. As an analogy, when the body is damaged (inflammation), the number of leukocytes increases and exceeds that in the healthy body. The elevation in the number of leukocytes can be considered a type of self-protective defense mechanism. Before the 20^th^ week of gestation, the MSP-α level remained relatively stable. An explanation for this finding is that the MSP-α level did not change with the gestational age, it changed according to the severity of the disease. Additionally, we found higher MSP expression (more intense MSP staining or more extensive staining) in the PE placentas compared to the normal control placentas, and placental MSP staining in the PE without severe features was significantly increased compared with that in the PE with severe features. These results indicate that the placental MSP level is associated with the development of PE and that MSP may be a beneficial factor for PE. These results are in agreement with the plasma analysis results. We also found that the placental expression of MSP receptor RON was positively correlated with the placental MSP expression, further indicating that MSP might have a certain role in the placenta.

Considering the above results, we speculate that an increased amount of MSP may be released from the placental trophoblast cells during pregnancy through proteolysis, giving rise to MSP-α. Thus, MSP may be a candidate protein for further analysis in studies of PE. Finally, we divided the PE patients into four subgroups: early onset without severe features, late onset without severe features, early onset with severe features and late onset with severe features. We also calculated the predictive efficacy of MSP-α in each of these groups. We found that MSP had higher predictive power for the PE without severe features group compared to the other groups, particularly for the early-onset PE without severe features.

Although MSP is primarily synthesized in the liver, the MSP/HGFL protein has been demonstrated to be present in the virgin and pregnant uteri [7]. In another study conducted by Degen, MSP/HGFL was found to be expressed at a lower level in the placenta [12], which was consistent with the results of this study. The presence of MSP and its receptor RON in the placenta has been previously demonstrated [6, 12], and we further confirmed this finding. Comoglio et al [13] have shown that MSP may promote the release of RON from the liver, adrenal glands and spinal ganglia in humans. Taken together, the results of this and previous studies suggest that MSP may be responsible for RON production in the placenta, which may account for the elevated MSP level in the placenta during the early stage of PE in pregnancy.

The assessment of altered MSP production and the comparative analysis of the implanted versus the non-implanted human blastocysts in relation to the development of PE have revealed that MSP plays a role in the pathogenesis of PE [14]. In another study, Bassett [15] has reported that MSP/MST1 may play a role in early development of the vertebrate placenta, which also suggests that MSP may be related to placental formation and may be involved in the occurrence of PE. However, the roles of MSP in these placental developmental processes and whether an increase in its level leads to the development of PE remain unknown. It has been previously demonstrated that MSP may have beneficial effects on the invasion of trophoblast cells and the implantation of the placenta via Ron [6, 7], which promotes the reconstruction of the uterine spiral artery and then alleviates the ischemia together with hypoxia of the placenta for preventing the excessive oxidative stress. These findings may be useful for predicting the prognoses of patients with PE and are in agreement with the results of our study. Thus, the complex interrelationship between MSP and RON may also be important in PE. Note that PE patients have increased the levels of soluble vascular endothelial growth factor receptor-1 (sFlt-1) and soluble endoglin (sEng) and a decreased level of placental growth factor (PIGF), as well as a decreased level of matrix metalloproteinases (MMP) and increased level of tissue inhibitors of metalloproteinase (TIMP), resulting in enhanced anti-angiogenic activities of sFlt-1/PIGF, decreased invasion activities of MMP/TIMP and abnormal functioning of endothelial cells [16, 17]. Further, increased MSP-α in preeclampsia may support the invasive function of RON. It is likely that some feedback mechanisms promote an increase in the MSP-α level to protect trophoblasts from apoptosis and to promote the invasion of trophoblast cells. MSP/RON signaling is a well-known process, particularly in tumors. It promotes the survival of tumor cells, cell invasion, and tumor angiogenesis [18, 19]. RON may contribute to the RAS–ERK pathway [5] and the PI3-K/AKT pathway [20], which regulate cell growth, survival and invasiveness [5]. It has also been demonstrated that insufficient RON production limits blastocyst development [6]. Preeclampsia has been associated with invasion of trophoblast cells and survival of endothelial cells, and it is generally believed that the shallow placental implantation in PE is the result of direct interference with trophoblast cell development. Hence, MSP/RON may play a role in the placental pathologic processes of PE.

In conclusion, MSP-α may be involved in the pathogenesis of PE. It is a protective factor and may be used as a biomarker in combination with other proteins (sFLT-1, etc.) to increase the predictive power for PE. However, future multicenter studies with larger samples must be performed to obtain accurate results regarding the early predictive value of MSP-α in combination with other factors, such as clinical risk factors and additional protein factors, for the clinical diagnosis of PE.

## Supporting Information

S1 Table patient characteristics of the placental samples. (DOC)

S2 Table Clinical characteristics of PE patients who are grouped in early onset PE without severe features, early onset PE with severe features, late onset PE without severe features and late onset PE with severe features. (DOC)

S1 Fig RON expression in trophoblast cells in placental sections from pregnant women, as determined by immunohistochemical staining. Placental expression of MSP receptor RON was positively correlated with placental MSP expression (DAB, brown: images E-G, 200X magnification), indicating that MSP might have a certain role in the placenta (images E: PE without severe features; images F: PE with severe features; images G: normal pregnancies in the control group).
(TIF)

S2 Fig MSP may play a role in preeclampsia, together with other protein factors. (TIF)

S1 Text The definition of the severe features of preeclampsia. (DOC)

S2 Text ELISA -Assays were performed in accordance with the manufacturer's instructions. (PDF)

S3 Text MSP antibody's instructions in immunohistochemistry assay. (PDF)

S4 Text RON antibody's instructions in immunohistochemistry assay. (PDF)
