# Supplementary material for: Plasma Level of Placenta-Derived Macrophage-Stimulating Protein -Chain in Preeclampsia before 20 Weeks of Pregnancy
Source: PLoS One. 2016 Aug 25;11(8):e0161626. doi: 10.1371/journal.pone.0161626 (PMC4999075; doi:10.1371/journal.pone.0161626)
Supplement: S1 Table — (DOC) [file pone.0161626.s001.doc]

S1 Table . patient characteristics of the placental samples.(**(A total of 17 PE, 15 early-onset and 2 late-onset and 9 severe feature, 8 without severe feature)**

| **Characteristic** | **Case（PE）,n=17** | **Control, n=17** | **P-Value** |
| --- | --- | --- | --- |
| Maternal Age(year) | 30.00±9.14 | 29.44±10.36 | 0.383 |
| Pre-pregnancy BMI (kg/m2) | 22.36±2.81 | 21.79±2.29 | 0.085 |
| delivery(week) | 34.88±6.86 | 36.29±4.38 | <0.001 |
| Systolic(mmHg) | 156.62±14.78 | 118.37±14.15 | <0.001 |
| Diastolic(mmHg) | 105.63±7.23 | 77.46±8.40 | <0.001 |
| Proteinuria(g/24h) | 3.21±4.65 | 0 | <0.05 |
| newborn-weight(g, n) | 2738.74±589.59 | 3034.56±280.33 | <0.001 |
| SGA newborns(n, ％) | 2（11.76％） | 0（0％） | <0.05 |
| FGR(n, ％) | 2( 11.76％) | 0（0％ | <0.05 |
| Cesarean section (n, ％) | 17（100％） | 1（5.88％） | <0.05 |
