# Supplementary material for: Plasma Level of Placenta-Derived Macrophage-Stimulating Protein -Chain in Preeclampsia before 20 Weeks of Pregnancy
Source: PLoS One. 2016 Aug 25;11(8):e0161626. doi: 10.1371/journal.pone.0161626 (PMC4999075; doi:10.1371/journal.pone.0161626)
Supplement: S2 Table — (DOC) [file pone.0161626.s002.doc]

**S2 Table :**

Clinical characteristics of PE patients who are grouped in early onset PE without severe features, early onset PE with severe features, late onset PE without severe features and late onset PE with severe features.

| **Characteristic** | Later onset & With severe features(n=16) | Later onset & Without severe features(n=27) | Early onset & With severe features(n=11) | Early onset & Without severe features(n=8) |
| --- | --- | --- | --- | --- |
| **Maternal Age(year)** | 28.69±4.19 | 29.78±4.01 | 30.55±4.37 | 32.00±5.76 |
| **Pre-pregnancy BMI (kg/m2)** | 22.07±2.44 | 21.54±2.95 | 20.15±3.19 | 21.88±2.35 |
| **MSP (ng /ml) before 20 weeks of gestation** | 123.19±46.72 | 195.23.84±46.32 | 168.39±46.46 | 237.35±59.11 |
| **Gestational age at delivery(week)** | 36.61±1.46 | 38.64±1.23 | 29.25±3.21 | 34.30±0.79 |
| **Systolic(mmHg)** | 164.31±8.44 | 142.96±9.55 | 167.64±9.71 | 147.00±11.84 |
| **Diastolic(mmHg)** | 109.56±7.83 | 94.81±6.29 | 106.91±7.37 | 97.50±7.07 |
| **Proteinuria(g/24h)** | 3.76±2.79 | 1.33±1.03 | 6.95±9.42 | 2.08±1.87 |
| **newborn-weight(g, n)** | 2498.13±417.02 | 3032.96±424.76 | 1150.45±538.95 | 2088.75±394.37 |
| **Cesarean or section Forceps delivery or induced labor (n, ％)** | 17（ 100％） | 9（33.3％） | 11（ 100％） | 8（ 100％） |
| **Stillbirth (n)** | 0 ( 0 ％) | 0 ( 0 ％) | 3 ( 27.27％) | 0 ( 0 ％) |
