# Supplementary figures and images for: Plasma Level of Placenta-Derived Macrophage-Stimulating Protein -Chain in Preeclampsia before 20 Weeks of Pregnancy
Source: PLoS One. 2016 Aug 25;11(8):e0161626. doi: 10.1371/journal.pone.0161626 (PMC4999075; doi:10.1371/journal.pone.0161626)

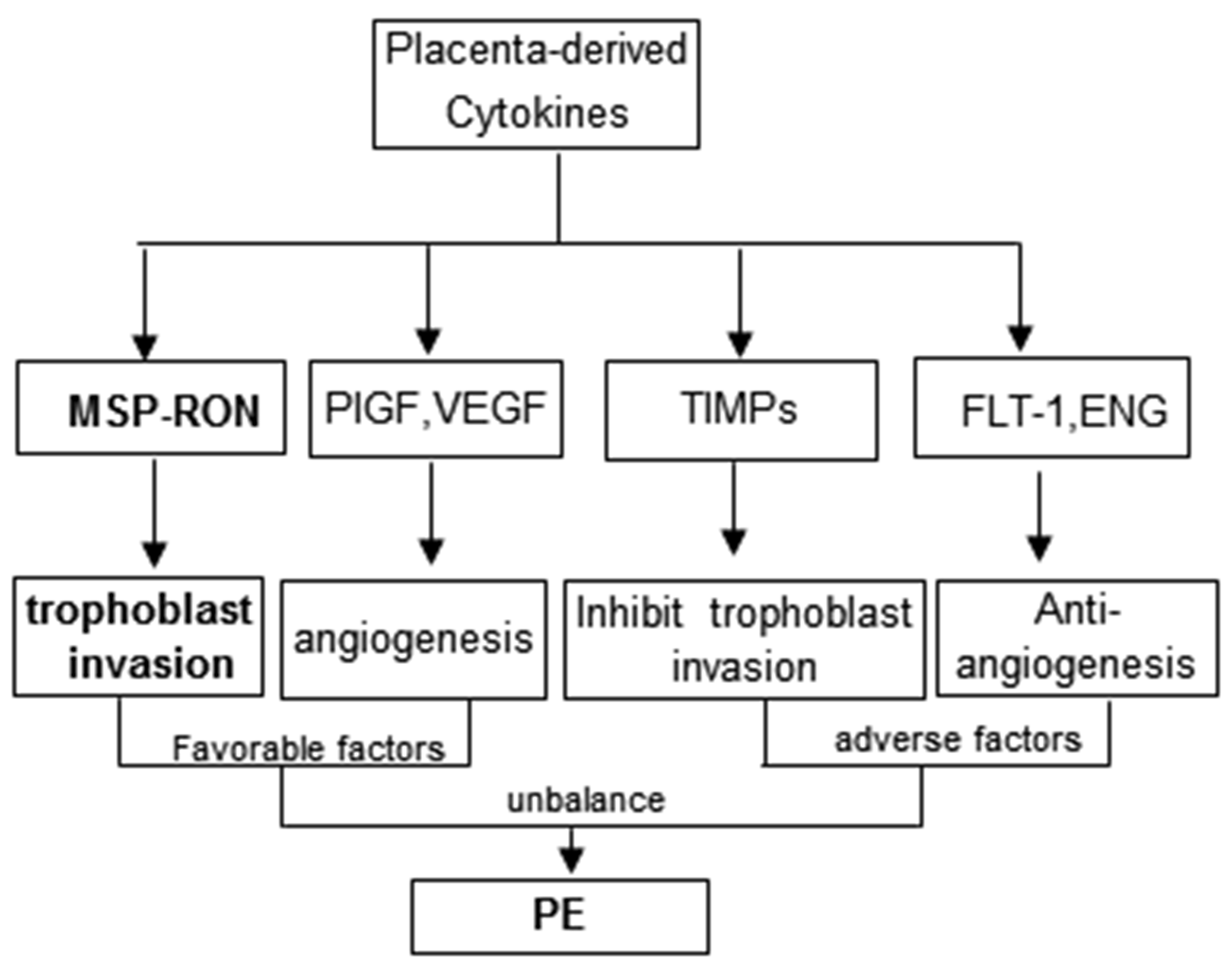

Supplement: S1 Fig — Placental expression of MSP receptor RON was positively correlated with placental MSP expression (DAB, brown: images E-G, 200X magnification), indicating that MSP might have a certain role in the placenta (images E: PE without severe features; images F: PE with severe features; images G: normal pregnancies in the control group). (TIF) [file pone.0161626.s003.tif]

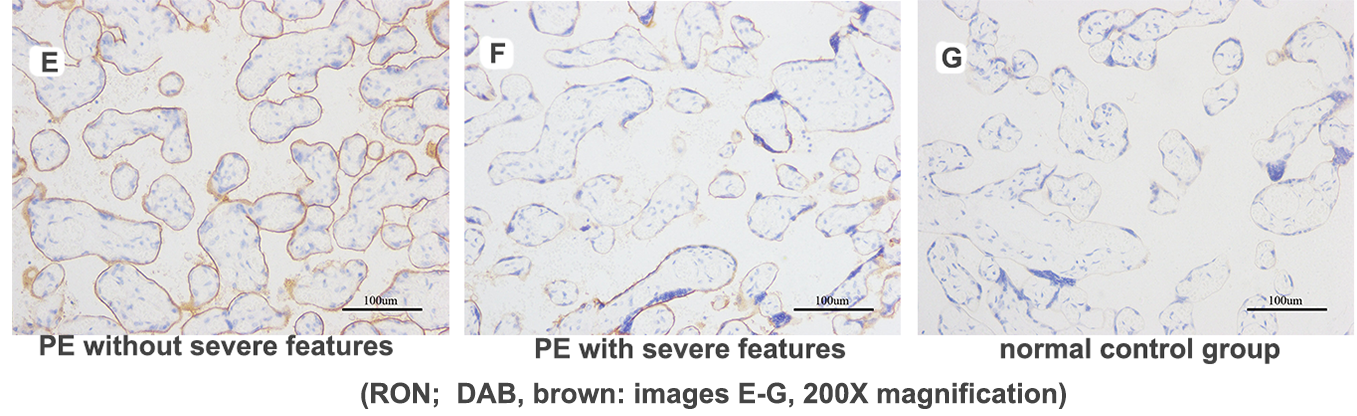

Supplement: S2 Fig — (TIF) [file pone.0161626.s004.tif]
