# Supplementary material for: Plasma Level of Placenta-Derived Macrophage-Stimulating Protein -Chain in Preeclampsia before 20 Weeks of Pregnancy
Source: PLoS One. 2016 Aug 25;11(8):e0161626. doi: 10.1371/journal.pone.0161626 (PMC4999075; doi:10.1371/journal.pone.0161626)
Supplement: S1 Text — (DOC) [file pone.0161626.s005.doc]

**S1 Text:**

**The severe features of preeclampsia**

**(1) systolic blood pressure ≥ 160mmhg or diastolic blood pressure≥ 110mmhg (two blood pressure measurements were taken at intervals of at least 4 hours when the patient is on bed rest); (2) thrombocytopenia (platelet < 100 x 10^9^ / L); (3)impaired liver function (serum transaminase levels was more than 2 times of normal value); (4) progressive renal insufficiency (serum creatinine greater than 1.1mg/dL or more than 2 times of normal values ); (5) pulmonary edema; (6) new onset cerebral or visual disturbances.**
