# Supplementary material for: Plasma Level of Placenta-Derived Macrophage-Stimulating Protein -Chain in Preeclampsia before 20 Weeks of Pregnancy
Source: PLoS One. 2016 Aug 25;11(8):e0161626. doi: 10.1371/journal.pone.0161626 (PMC4999075; doi:10.1371/journal.pone.0161626)
Supplement: S2 Text — (PDF) [file pone.0161626.s006.pdf]

RayBio® Custom *SpeedE*LISA Kit

Catalog #: ELXS-PRELIM

User Manual
Last revised February 19, 2016

Caution:
Extraordinarily useful information enclosed

RayBiotech
The protein array pioneer

ISO 13485 Certified

3607 Parkway Lane, Suite 100
Norcross, GA 30092
Tel: 1-888-494-8555 (Toll Free) or 770-729-2992, Fax:770-206-2393
Web: www.RayBiotech.com, Email: info@raybiotech.com

RayBiotech, Inc.

RayBio® Custom *SpeedE*LISA Protocol

Table of Contents

Section		Page #
**I.**	Introduction	3
**II.**	How It Works	3
**III.**	Storage	4
**IV.**	Reagents	4
**V.**	Additional Materials Required	4
**VI.**	Reagent Preparation	5
**VII.**	Assay Procedure	6
**VIII.**	Assay Procedure Summary	7
**IX.**	Calculation of Results	8
	A. Typical Data	8
	B. Sensitivity	8
	C. Spiking & Recovery	9
	D. Linearity	9
	E. Reproducibility	10
**X.**	Specificity	10
**XI.**	Troubleshooting Guide	11

Please read the entire manual carefully before starting your experiment

I. INTRODUCTION

The RayBio Custom ***SpeedE***LISA kit is an in vitro enzyme-linked immunosorbent assay for the quantitative measurement of target protein in biological sample such as serum, plasma, and cell culture supernatants*. This ***SpeedE***LISA employs a biotinylated capture and a HRP-conjugated detection antibody which immunocaptures the sample analyte in solution. The microplate in the kit is pre-coated with streptavidin. The biotinylated capture antibody/protein/HRP-conjugated detection antibody mixture is pipetted into the wells and the biotinylated target protein present in a sample is bound to the wells by the immobilized streptavidin. After incubation, the wells are washed to remove unbound material.

A TMB substrate solution is added to the wells and color develops in proportion to the amount of target protein bound. The Stop Solution changes the color from blue to yellow, and the intensity of the color is measured at 450 nm.

***Note:** Biotin interferes with this assay. If your samples contain biotin (commonly found in certain cell culture media such as RPMI, 1640, or F-12K), we recommend using the standard RayBio Sandwich ELISA kit.

<u>What does it mean when a kit is list as 'Custom'?</u>

SpeedELISA kits that are listed as 'custom' require a 6-8 week development phase, which RayBiotech begins after receiving an order for the kit. The development only needs to occur once, after which the final kit will have passed Raybiotech's ELISA quality control tests, and will thereafter be a stock kit with normal lead time.

II. How It Works

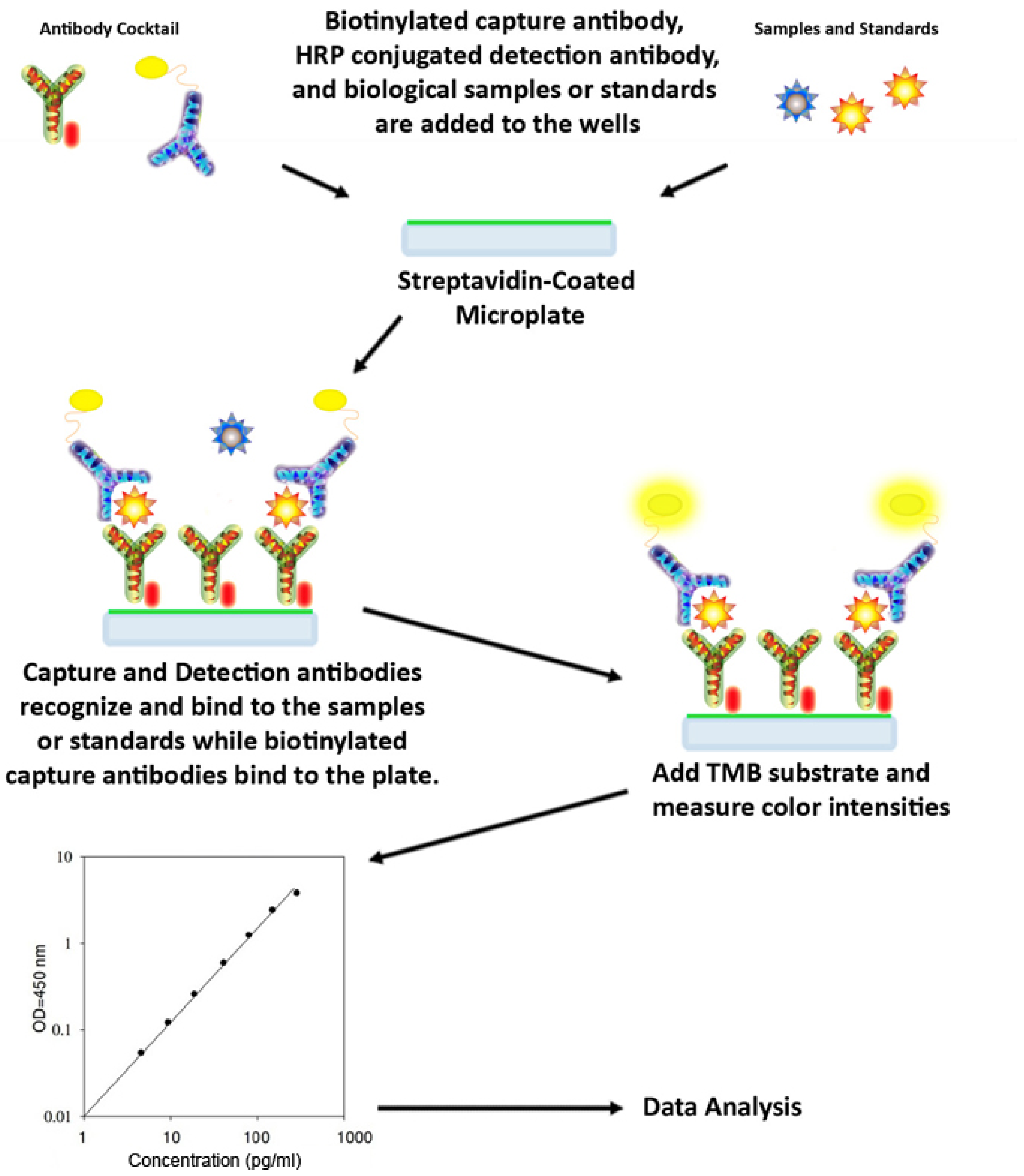

III. STORAGE

The entire kit may be stored at -20°C for up to 1 year from the date of shipment. Avoid repeated freeze-thaw cycles. The kit may be stored at 4°C for up to 6 months. For extended storage, it is recommended to store at -80°C. For prepared reagent storage, see table below.

IV. REAGENTS

Component	Size / Description	Storage / Stability After Preparation
Streptavidin-Coated Microplate (Item A)	96 wells (12 strips x 8 wells) coated with streptavidin.	1 month at 4°C*
Wash Buffer Concentrate (20X) (Item B)	25 ml of 20X concentrated solution.	1 month at 4°C
Standard Protein (Item C)	2 vials of target protein. 1 vial is enough to run each standard in duplicate.	1 week at -80°C
Biotinylated-Conjugated Capture Antibody (Item F)	2 vial of Biotinylated anti- target protein. 1 vial is enough to assay half microplate.	5 days at 4°C
HRP Conjugated Detection Antibody (Item G)	2 vials of HRP conjugated anti- target protein. 1 vial is enough to assay half the microplate.	5 days at 4°C
TMB One-Step Substrate Reagent (Item H)	12 ml of 3,3,5,5'-tetramethylbenzidine (TMB) in buffer solution.	N/A
Stop Solution (Item I)	8 ml of 0.2 M sulfuric acid.	N/A
Assay Diluent	15 ml of 5X concentrated buffer	1 month at 4°C

*Return unused wells to the pouch containing desiccant pack, reseal along entire edge.

V. ADDITIONAL MATERIALS REQUIRED

1. Microplate reader capable of measuring absorbance at 450 nm.
2. Precision pipettes to deliver 2 µl to 1 ml volumes.
3. Adjustable 1-25 ml pipettes for reagent preparation.
4. 100 ml and 1 liter graduated cylinders.
5. Absorbent paper.
6. Distilled or deionized water.
7. Log-log graph paper or computer and software for ELISA data analysis.
8. Tubes to prepare standard or sample dilutions.

VI. REAGENT PREPARATION

1. Bring all reagents and samples to room temperature (18 - 25ºC) before use.

2. Assay Diluents should be diluted 5-fold with deionized or distilled water before use.

3. Briefly spin down the biotinylated Capture Antibody vial (Item F) before use. Add 1.9 ml of 1X Assay Diluent into the vial to prepare the capture antibody solution. Pipette up and down to mix gently. This capture antibody solution will be used in step 5 of Part VI: Reagent Preparation.

4. Briefly spin down the HRP-conjugated Detection Antibody vial (Item G) before use. Add 1.9 ml of 1X Assay Diluent into the vial to prepare the detection antibody solution. Pipette up and down to mix gently. This detection antibody solution will be used in the following step.

5. Prepare the Antibody Cocktail: a 1:1 mixture of the biotinylated capture antibody and the HRP-conjugated detection antibody. This antibody cocktail will be used in step 3 of Part VII: Assay Procedure.
   For example: Add 1.7 ml of prepared biotinylated capture antibody solution and 1.7 ml HRP-conjugated detection antibody solution into a tube. This yields 3.4 ml of Antibody Coctail, enough to assay half the plate. Mix well and use immediately.

6. Preparation of Standards: Appropriate standard preparation will be determined during the development process.

7. Sample Dilution: Dilute samples using provided Assay Diluent(s).
   Note: Levels of target protein may vary between different specimens. Optimal dilution factors for each sample must be determined by the investigator.

8. If the Wash Concentrate (20X) (Item B) contains visible crystals, warm to room temperature and mix gently until dissolved. Dilute 20 ml of Wash Buffer Concentrate into deionized or distilled water to yield 400 ml of 1X Wash Buffer.

VII. ASSAY PROCEDURE

1. Bring all reagents and samples to room temperature (18 - 25ºC) before use. It is recommended that all standards and samples be run at least in duplicate.

2. Label removable 8-well strips as appropriate for your experiment.

3. Add 70 µl of the Antibody Cocktail (see Reagent Preparation step 5) into appropriate wells. Cover wells and incubate for 10 minutes at room temperature.

4. Add 50 µl of standard (see Reagent Preparation step 6) or each sample (see Reagent Preparation step 7) into each well with the Antibody Cocktail added in Step 3. Cover wells and incubate for 2 hours at room temperature.

5. Discard the solution and wash 4 times with 1x Wash Solution. Wash by filling each well with Wash Buffer (300 µl) using a multi-channel Pipette or autowasher. Complete removal of liquid at each step is essential to good performance. After the last wash, remove any remaining Wash Buffer by aspirating or decanting. Invert the plate and blot it against clean paper towels.

6. Add 100 µl of TMB One-Step Substrate Reagent (Item H) to each well. Incubate for 30 minutes at room temperature in the dark with gentle shaking.

7. Add 50 µl of Stop Solution (Item I) to each well. Read at 450 nm immediately.

VIII. ASSAY PROCEDURE SUMMARY

1. Prepare all reagents, samples and standards as instructed.

2. Add 70 µl of the Antibody Cocktail to the appropiate wells and incubate for 10 minutes at room temperature.

3. Add 50 µl of sample or standard to each well and incubate for 2 hours at room temperature.

4. Wash wells.

5. Add 100 µl of TMB One-Step Substrate Reagent to each well. Incubate for 30 minutes at room temperature.

6. Add 50 µl of Stop Solution to each well. Read at 450 nm immediately.

IX. CALCULATION OF RESULTS

Calculate the mean absorbance for each set of duplicate standards, controls and samples, and subtract the average zero standard optical density. Plot the standard curve on log-log graph paper or using Sigma plot software, with standard concentration on the x-axis and absorbance on the y-axis. Draw the best-fit straight line through the standard points.

A. TYPICAL DATA

An example of a typical standard curve for this kit will be avaliable after the development process.

B. SENSITIVITY

The minimum detectable dose of the target protein will by determined during the development process.

Minimum dectable dose is define as the analyte concentration resulting in an absorbance that is 2 standard deviation higher than that of the blank (diluent buffer).

C.RECOVERY

Recovery will be determined during the development process by spiking various levels of the target protein into serum, plasma and cell culture media.

D. LINEARITY

Linearity will be determined in serum, plasma and cell culture media during the development process

E. REPRODUCIBILITY

Intra-Assay CV%: <10%

Inter-Assay CV%: <12%

X. SPECIFICITY

Specificity will be determined during the development process.

XI. TROUBLESHOOTING GUIDE

Problem	Cause	Solution
Poor standard curve	• Inaccurate pipetting<br>• Improper standard dilution	• Check pipettes<br>• Briefly centrifuge Item C and dissolve the powder thoroughly by gently mixing
Low signal	• Improper preparation of standard and/or biotinylated antibody (Item G)<br>• Too brief incubation times<br>• Inadequate reagent volumes or improper dilution	• Briefly spin down vials before opening. Dissolve the powder thoroughly.<br>• Ensure sufficient incubation time; assay procedure step 2 may be done overnight<br>• Check pipettes and ensure correct preparation
Large CV	• Inaccurate pipetting<br>• Air bubbles in wells	• Check pipettes<br>• Remove bubbles in wells
High background	• Plate is insufficiently washed<br>• Contaminated wash buffer	• Review the manual for proper wash. If using a plate washer, ensure that all ports are unobstructed.<br>• Make fresh wash buffer
Low sensitivity	• Improper storage of the ELISA kit<br>• Stop solution	• Store your standard at <-70ºC after reconstitution, others at 4ºC. Keep substrate solution protected from light.<br>• Add stop solution to each well before reading plate

RayBio® ELISA Kits

Over 2,000 ELISA kits available, visit www.RayBiotech.com/ELISA-Kits.html for details.

This product is for research use only.

©2015 RayBiotech, Inc
