# Supplementary material for: Plasma Level of Placenta-Derived Macrophage-Stimulating Protein -Chain in Preeclampsia before 20 Weeks of Pregnancy
Source: PLoS One. 2016 Aug 25;11(8):e0161626. doi: 10.1371/journal.pone.0161626 (PMC4999075; doi:10.1371/journal.pone.0161626)
Supplement: S3 Text — (PDF) [file pone.0161626.s007.pdf]

abcam

Product datasheet

Anti-MST1 antibody [EPR6207] ab124787

RabMAb®

2 图像

概述

产品名称	Anti-MST1抗体[EPR6207]
描述	兔单克隆抗体[EPR6207] to MST1
经测试应用	WB, IHC-P, Flow Cyt
种属反应性	与反应: Mouse, Rat, Human
免疫原	Synthetic peptide corresponding to residues in Human MST1.
阳性对照	HepG2, 293T and Caco-2 cell lysates. Human liver carcinoma tissue.
常规说明	This product is a recombinant rabbit monoclonal antibody.<br>Produced using Abcam's RabMAb® technology. RabMAb® technology is covered by the following U.S. Patents, No. 5,675,063 and/or 7,429,487.

性能

形式	Liquid
存放说明	Shipped at 4°C. Store at -20°C. Stable for 12 months at -20°C.
存储溶液	PBS 49%,Sodium azide 0.01%,Glycerol 50%,BSA 0.05%
纯度	Tissue culture supernatant
克隆	单克隆
克隆编号	EPR6207
同种型	IgG

应用

Our Abpromise guarantee covers the use of **ab124787** in the following tested applications.

The application notes include recommended starting dilutions; optimal dilutions/concentrations should be determined by the end user.

应用	Abreviews	说明
WB		1/1000 - 1/10000. Detects a band of approximately 85 kDa (predicted molecular weight: 80 kDa).

应用	Abreviews	说明
IHC-P		1/50 - 1/100. Perform heat mediated antigen retrieval before commencing with IHC staining protocol.
Flow Cyt		1/10 - 1/100. ab172730-Rabbit monoclonal IgG, is suitable for use as an isotype control with this antibody.

应用说明	Is unsuitable for ICC or IP.

靶标

功能	Probably has no proteolytic activity, since crucial characteristic of serine proteases catalytic sites are not conserved.
序列相似性	Belongs to the peptidase S1 family. Plasminogen subfamily.<br>Contains 4 kringle domains.<br>Contains 1 PAN domain.<br>Contains 1 peptidase S1 domain.
翻译后修饰	May be cleaved after Arg-483, to yield two chains held together by disulfide bonds, or two separate polypeptides.
细胞定位	Secreted.

Anti-MST1 antibody [EPR6207] 图像

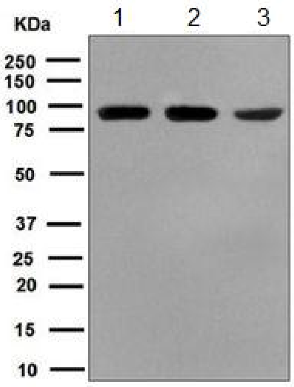

Western blot - Anti-MST1 antibody [EPR6207] (ab124787)

All lanes : Anti-MST1 antibody [EPR6207] (ab124787) at 1/1000 dilution

Lane 1 : HepG2 cell lysate
Lane 2 : 293T cell lysate
Lane 3 : Caco-2 cell lysate

Lysates/proteins at 10 µg per lane.

Secondary
Standard HRP labelled goat anti-rabbit at 1/2000 dilution

Predicted band size : 80 kDa
Observed band size : 85 kDa

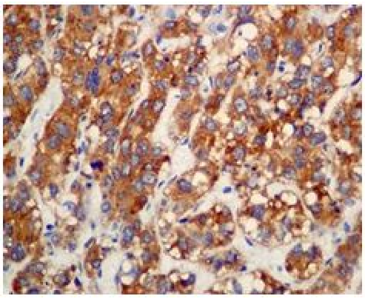

Immunohistochemistry (Formalin/PFA-fixed paraffin-embedded sections) - Anti-MST1 antibody [EPR6207] (ab124787)

ab124787 at 1/50 dilution staining MST1 in paraffin-embedded Human liver carcinoma tissues by Immunohistochemistry.

Please note: All products are "FOR RESEARCH USE ONLY AND ARE NOT INTENDED FOR DIAGNOSTIC OR THERAPEUTIC USE"

Our Abpromise to you: Quality guaranteed and expert technical support

- Replacement or refund for products not performing as stated on the datasheet
- Valid for 12 months from date of delivery
- Response to your inquiry within 24 hours

- We provide support in Chinese, English, French, German, Japanese and Spanish
- Extensive multi-media technical resources to help you
- We investigate all quality concerns to ensure our products perform to the highest standards

If the product does not perform as described on this datasheet, we will offer a refund or replacement. For full details of the Abpromise, please visit http://www.abcam.cn/abpromise or contact our technical team.

Terms and conditions

- Guarantee only valid for products bought direct from Abcam or one of our authorized distributors
