# Supplementary material for: Plasma Level of Placenta-Derived Macrophage-Stimulating Protein -Chain in Preeclampsia before 20 Weeks of Pregnancy
Source: PLoS One. 2016 Aug 25;11(8):e0161626. doi: 10.1371/journal.pone.0161626 (PMC4999075; doi:10.1371/journal.pone.0161626)
Supplement: S4 Text — (PDF) [file pone.0161626.s008.pdf]

abcam

Product datasheet

Anti-RON antibody [EP1132Y] ab52927

RabMAb®

1 Abreviews | 1 References | 3 图像

概述

产品名称	Anti-RON抗体[EP1132Y]
描述	兔单克隆抗体[EP1132Y] to RON
经测试应用	WB, IP, Flow Cyt, IHC-P, ICC/IF
种属反应性	与反应: Human
免疫原	A synthetic peptide corresponding to residues near the N-terminus of human RON.
阳性对照	SKBR3 cell lysate; human stomach tissue.
常规说明	This product is a recombinant rabbit monoclonal antibody.<br><br>Produced using Abcam's RabMAb® technology. RabMAb® technology is covered by the following U.S. Patents, No. 5,675,063 and/or 7,429,487. The major RON transcript is translated into a glycosylated single chain precursor, cleaved into a 185 kDa heterodimer (p185Ron) of 35 (alpha) and 150 kDa (beta) disulfide-linked chains, before exposure at the cell surface.<br><br>Mouse, Rat: We have preliminary internal testing data to indicate this antibody may not react with these species. Please contact us for more information.

性能

形式	Liquid
存放说明	Shipped at 4°C. Store at -20°C. Stable for 12 months at -20°C.
存储溶液	PBS 49%,Sodium azide 0.01%,Glycerol 50%,BSA 0.05%
纯度	Tissue culture supernatant
克隆	单克隆
克隆编号	EP1132Y
同种型	IgG

应用

Our Abpromise guarantee covers the use of **ab52927** in the following tested applications.

The application notes include recommended starting dilutions; optimal dilutions/concentrations should be determined by the end user.

应用	Abreviews	说明
WB		1/10000. Detects a band of approximately 35,150,180 kDa (predicted molecular weight: 185 kDa). 1/10000. Detects a band of approximately 35/150/180 kDa (predicted molecular weight: 185 kDa). See Notes section of Datasheet.
IP		1/30.
Flow Cyt		1/20 - 1/40. (methanol fixed cells)<br><br>ab172730-Rabbit monoclonal IgG, is suitable for use as an isotype control with this antibody.
IHC-P	★★★☆☆	Use at an assay dependent concentration.
ICC/IF		1/50 - 1/100.

靶标

功能	Receptor for macrophage stimulating protein (MSP). Has a tyrosine-protein kinase activity.
组织特异性	Keratinocytes and lung.
序列相似性	Belongs to the protein kinase superfamily. Tyr protein kinase family.<br>Contains 3 IPT/TIG domains.<br>Contains 1 protein kinase domain.<br>Contains 1 Sema domain.
翻译后修饰	Proteolytic processing yields the two subunits.<br>Phosphorylated in response to ligand binding.
细胞定位	Membrane.

Anti-RON antibody [EP1132Y] 图像

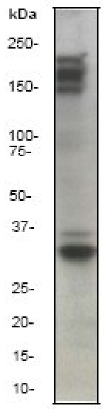

Western blot - RON antibody [EP1132Y] (ab52927)

Anti-RON antibody [EP1132Y] (ab52927) at 1/10000 dilution + SKBR3 cell lysate at 10 µg

Secondary

HRP-labelled goat anti-rabbit

Predicted band size : 185 kDa

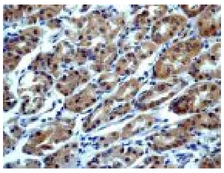

Immunohistochemistry (Paraffin-embedded sections) - RON antibody [EP1132Y] (ab52927)

Immunohistochemical staining of paraffin-embedded human stomach tissue using ab52927 at a dilution of 1/100-1/250.

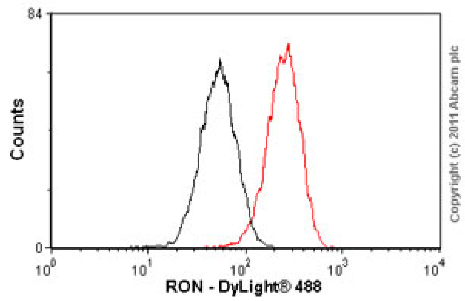

Flow Cytometry - RON antibody [EP1132Y] (ab52927)

Overlay histogram showing THP1 cells stained with ab52927 (red line). The cells were fixed with methanol (5 min) and incubated in 1x PBS / 10% normal goat serum / 0.3M glycine to block non-specific protein-protein interactions. The cells were then incubated with the antibody (ab52927, 1/20 dilution) for 30 min at 22ºC. The secondary antibody used was DyLight® 488 goat anti-rabbit IgG (H+L) (ab96899) at 1/500 dilution for 30 min at 22ºC. Isotype control antibody (black line) was rabbit monoclonal IgG (1μg/1x10^6 cells) used under the same conditions. Acquisition of >5,000 events was performed. This antibody gave a decreased signal in THP1 cells fixed with 4% paraformaldehyde (10 min) used under the same conditions.

Please note that Abcam do not have any data for use of this antibody in non-fixed cells. We welcome any customer feedback.

Please note: All products are "FOR RESEARCH USE ONLY AND ARE NOT INTENDED FOR DIAGNOSTIC OR THERAPEUTIC USE"

Our Abpromise to you: Quality guaranteed and expert technical support

- Replacement or refund for products not performing as stated on the datasheet
- Valid for 12 months from date of delivery
- Response to your inquiry within 24 hours

- We provide support in Chinese, English, French, German, Japanese and Spanish
- Extensive multi-media technical resources to help you
- We investigate all quality concerns to ensure our products perform to the highest standards

If the product does not perform as described on this datasheet, we will offer a refund or replacement. For full details of the Abpromise, please visit http://www.abcam.cn/abpromise or contact our technical team.

Terms and conditions

- Guarantee only valid for products bought direct from Abcam or one of our authorized distributors
